# Phosphonated Ionomers of Intrinsic Microporosity with Partially Ordered Structure for High-Temperature Proton Exchange Membrane Fuel Cells

**DOI:** 10.1021/acscentsci.3c00146

**Published:** 2023-03-16

**Authors:** Xi Sun, Jiayu Guan, Xue Wang, Xiaofeng Li, Jifu Zheng, Shenghai Li, Suobo Zhang

**Affiliations:** † Key Laboratory of Polymer Ecomaterials, Changchun Institute of Applied Chemistry, Chinese Academy of Sciences, Changchun 130022, China; ‡ University of Science and Technology of China, Hefei 230026, China

## Abstract

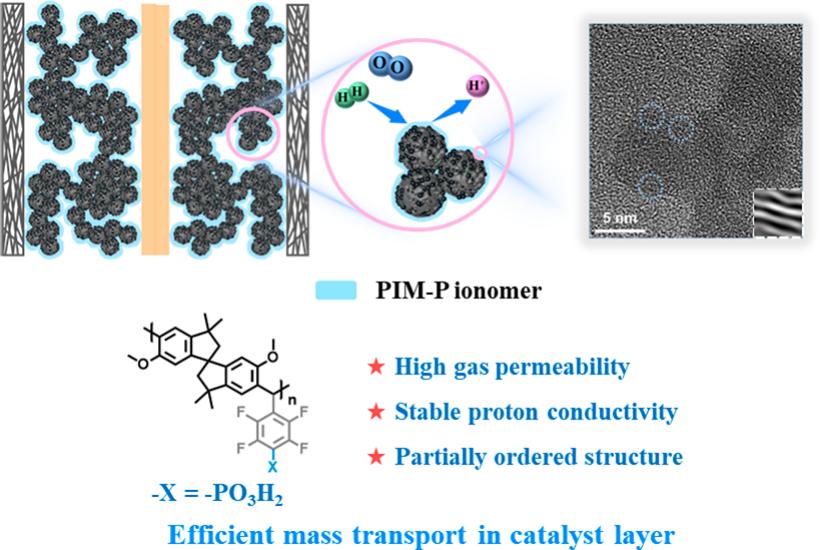

High mass transport resistance within the catalyst layer is one of the major factors restricting the performance and low Pt loadings of proton exchange membrane fuel cells (PEMFCs). To resolve the issue, a novel partially ordered phosphonated ionomer (PIM-P) with both an intrinsic microporous structure and proton-conductive functionality was designed as the catalyst binder to improve the mass transport of electrodes. The rigid and contorted structure of PIM-P limits the free movement of the conformation and the efficient packing of polymer chains, resulting in the formation of a robust gas transmission channel. The phosphonated groups provide sites for stable proton conduction. In particular, by incorporating fluorinated and phosphonated groups strategically on the local side chains, an orderly stacking of molecular chains based on group assembly contributes to the construction of efficient mass transport pathways. The peak power density of the membrane electrode assembly with the PIM-P ionomer is 18–379% greater than that of those with commercial or porous catalyst binders at 160 °C under an H_2_/O_2_ condition. This study emphasizes the crucial role of ordered structure in the rapid conduction of polymers with intrinsic microporosity and provides a new idea for increasing mass transport at electrodes from the perspective of structural design instead of complex processes.

## Abstract 2

A partially ordered phosphonated ionomer with both intrinsic microporous structure and proton-conductive functionality is disclosed as a catalyst binder to achieve efficient mass transport of catalyst layers in high-temperature proton exchange membrane fuel cells.

## Introduction

Fuel cells (FCs) are a type of electrochemical device that directly converts the chemical energy of fuels into electrical energy, independent of the Carnot cycle, and considered to be a promising clean and highly efficient technology.^1,2^ High-temperature proton exchange membrane fuel cells (HT-PEMFCs) generally operate in the range of 120–250 °C,^3^ which provides many specific advantages including faster reaction kinetics at the electrodes, enhanced tolerance to CO, and better heat and water management.^3−6^ Membrane electrode assemblies (MEAs) are vital components of PEMFCs where electrochemical reactions take place and are composed of proton exchange membranes (PEMs), catalyst layers (CLs), and gas diffusion layers (GDLs).^7−9^ Optimizing the composition of MEAs enhances the efficiency of FCs.^10^ Over the past few decades, researchers around the world have been focusing on developing a wide variety of new materials, including catalysts for oxygen reduction and PEMs with high proton conductivity.^11−16^ Recently, ionomers have attracted extensive attention as one of the main components of MEAs, which strongly affects the morphology and efficiency of CLs.

On the one hand, ionomers connect catalysts and PEMs, which can boost the integral interface between them. On the other hand, as the dispersant of catalysts, ionomers have great significance for improving the electrochemical triple-phase boundary (TPB) for the conduction of gases (reactants), ions, and electrons.^17−20^ Compared with PEMs, where no or much less gas permeability is required to limit the crossover of fuels (hydrogen and oxygen gases), ionomers should be gas-permeable so that those gaseous fuels can reach the reaction sites on the catalysts promptly.^21,22^ They overcome the constraints of the mass transport of reactants and facilitate the transportation of essential substances for redox reactions. Meanwhile, ionomers are also a direct physical barrier against the coalescence and detachment of catalysts.^23^ In other words, ideal ionomers will enhance the utilization and stability of catalysts, which are critical in achieving efficient mass transport and low Pt loadings in FCs.

Commercially available ionomers are mainly perfluorinated sulfonic acid (PFSA) ionomers with a poly(tetrafluoroethylene) (PTFE) backbone. These ionomers show remarkable proton conductivity and mechanical strength under fully hydrated conditions, but serious mass transport losses occur in the electrodes due to poor gas permeability.^24−26^ PTFE is often used as a catalyst binder in high-temperature PEMFCs (HT-PEMFCs) because of its excellent hydrophobicity, thermal stability, mechanical strength, and microporous structure formed after heat treatment, but there are also the drawbacks of a lack of proton conductivity and partial crystalline structure.^27,28^ The groups of Kim^29^ and Jannasch^30^ designed and synthesized phosphonated poly(pentafluorostyrene) and poly(arylene perfluorophenylphosphonic acid), respectively, which could conduct protons inherently under both hydrated and anhydrous conditions. Moreover, they found that due to the existence of the strong electron-withdrawing group (fluorophenyl), pentafluorophenylphosphonic acid had much better thermal stability than other types of phosphoric acids. In particular, phosphonated poly(pentafluorostyrene) exhibited stable proton conductivity even at 200 °C. However, ameliorating gas diffusion through binders, especially oxygen accessibility, is still a major challenge in HT-PEMFCs.^31^ Recently, great efforts have been devoted to promoting gas permeation in ionomers. Modestino et al.^22^ reported that the amorphous domain with a high fractional free volume (FFV) in PFSA ionomers reduced the resistance for gas permeation significantly. Kim and co-workers^29,31^ investigated the dispersing-agent-induced phase separation to fabricate the porous structure in phosphonated poly(pentafluorostyrene) ionomers. Wang et al.^32^ presented a composite ionomer by introducing a sulfonated covalent organic framework (COF) into Nafion to promote oxygen permeation. However, the preparation of ionomers with oxygen accessibility, proton conductivity, and interfacial compatibility via rational structural design is a huge challenge.

**Figure 1:**
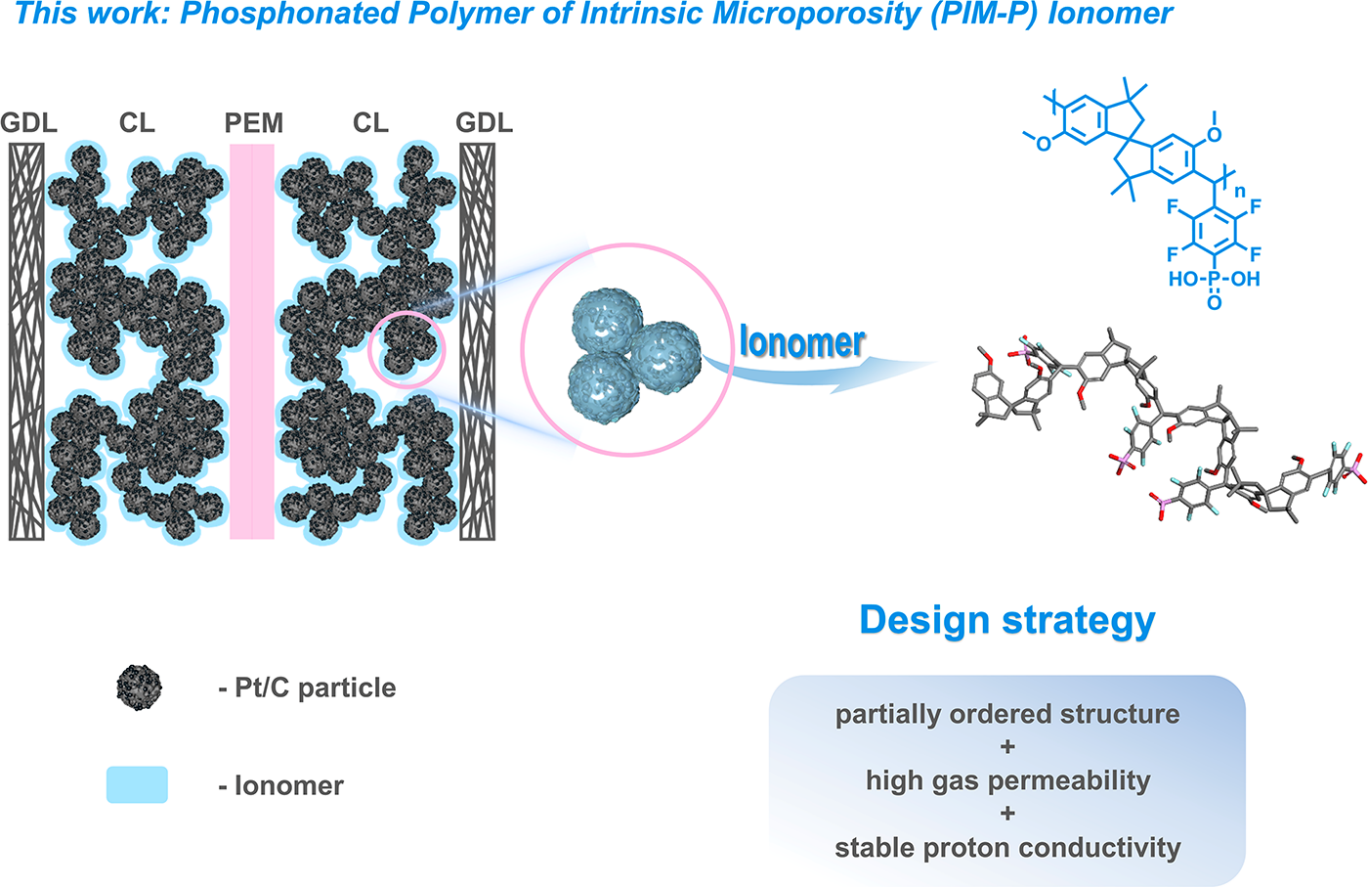
Design ideas of ionomers in PEMFCs.

Polymers of intrinsic microporosity (PIMs) are an emerging class of amorphous porous polymers. Due to the rigid and contorted molecular structures, which restrict the efficient packing of chains, PIMs have been widely studied based on solution-processing and ultrapermeable characteristics.^33,34^ The microporous structure of PIMs can be adjusted and modified by several methods including (i) tuning the angle of contorted centers, (ii) introducing pendant groups, or (iii) cross-linking of molecular chains.^35^ In recent years, researchers have attempted to introduce the rigid and contorted molecular structures into ionomers to improve the mass transport of low-temperature polymer electrolyte membrane fuel cells.^36−38^ However, in the harsh environment of HT-PEMFCs, there is still a lack of efficient ionomers with stable structures. Here, we present a novel phosphonated ionomer (PIM-P) with both an intrinsic microporous structure and proton-conductive functionality to decrease the mass transport resistance (Figure 1). PIM-P has inherent micropores to form a robust gas transmission channel, while the −PO_3_H_2_ groups provide stable proton conduction sites. Further, the SBI (spirobisindane) units reduce the phenyl contents of the backbone, which naturally alleviates defective phenyl group adsorption.^39,40^ Importantly, in contrast to amorphous polymers, PIM-P shows a unique partially ordered structure due to the assembly of the −PO_3_H_2_ groups that promote the orderly stacking of molecular chains. The ordered structure facilitated the stable conduction of gases and protons to construct ideal mass transport pathways, thus accelerating redox reactions, especially the oxygen reduction reaction (ORR) in the cathode. Given these benefits, the MEA with the PIM-P ionomer exhibited better electrode performance and higher peak power density than other commercial or porous binders.

## Result and Discussion

**Figure 2:**
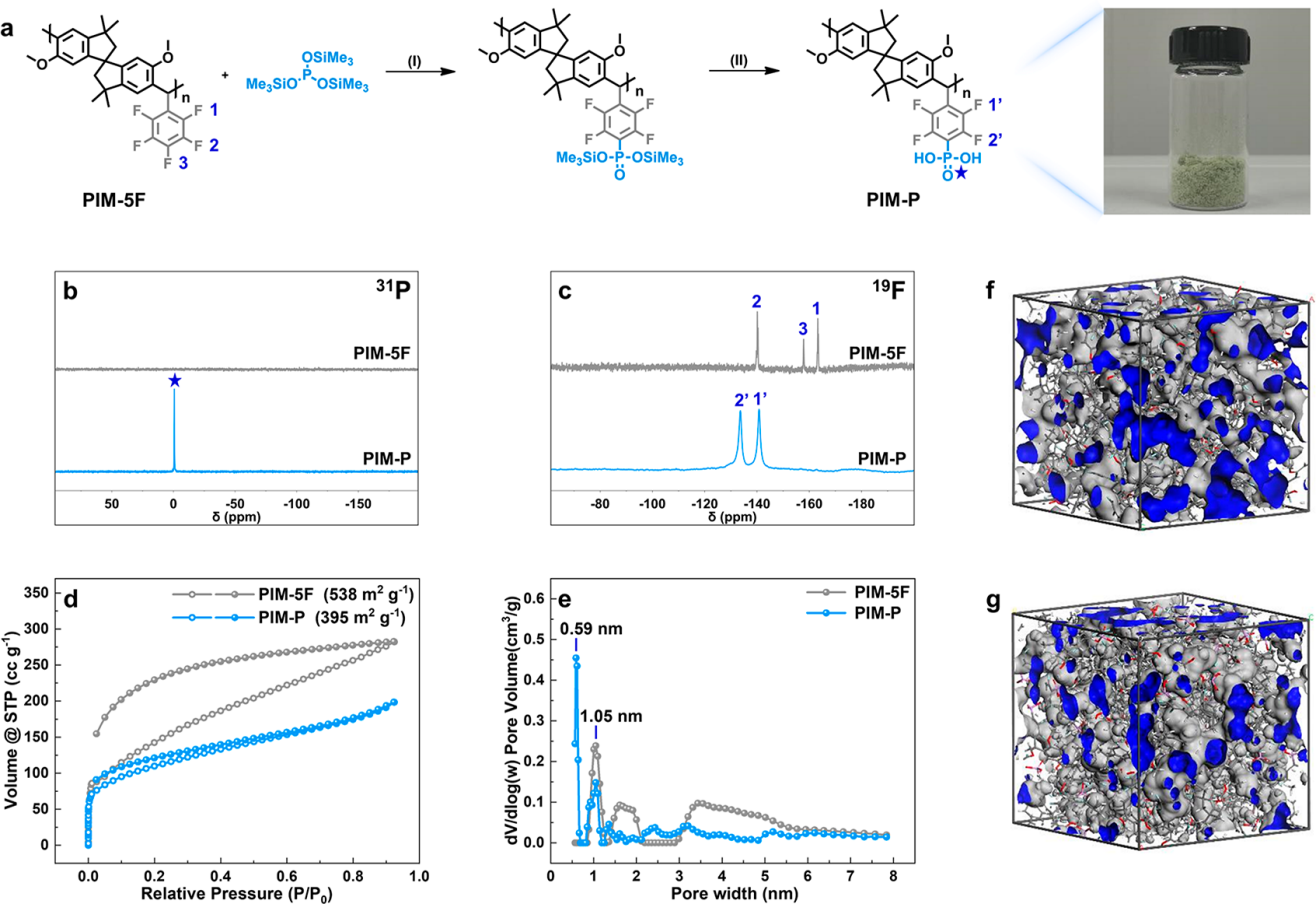
(a) Synthetic pathway to phosphonated poly(pentafluorophenylalkane): (I) phosphonation (DMAc, 190 °C, 12 h) and (II) hydrolysis (1 M HCl, 110 °C, 12 h); ^31^P (b) and ^19^F (c) NMR spectra of PIM-P and PIM-5F; (d) N_2_ adsorption/desorption isotherms of PIM-P and PIM-5F at 77 K; (e) pore size distributions of them from N_2_ sorption isotherms; three-dimensional view of PIM-5F (f) and PIM-P (g) modeling structure in an amorphous cell.

As shown in Figure 2a, PIM-P was synthesized via a nucleophilic substitution reaction. Using DMSO-*d*_6_ or CDCl_3_ as a solvent, the structure of PIM-P was determined by ^31^P, ^19^F, and ^1^H nuclear magnetic resonance (NMR). Based on the ^31^P NMR spectrum of PIM-P (Figure 2b), a single signal assigned to −PO_3_H_2_ at −0.81 ppm indicated successful phosphonation. Compared with the three peaks with an integral ratio of 2:1:2 in the ^19^F NMR spectrum of PIM-5F, two new peaks at −133.7 and −140.9 ppm with an equal ratio were detected in the spectrum of PIM-P (Figure 2c). It demonstrated that the substitution reaction selectively occurred at the para position of the tertiary carbon and the conversion to phosphonic acid group was complete. The substitution reaction was also confirmed by the appearance of a few new bands in the Fourier transform infrared (FT-IR) spectra (Figure S3). Compared with PIM-5F, the absorption bands at 1456 and 555 cm^–1^ in the spectrum of PIM-P were assigned to the stretching and bending vibrations of PO_3_, respectively. The absorption bands at 1043 and 1271 cm^–1^ were assigned to the stretching vibrations of P–O and P=O, respectively. The FT-IR spectrum of PIM-P also showed relatively broad and weak bands associated with the stretching vibration of the hydrogen bonds (H-bonds) in (P)O–H at 2400–3600 cm^–1^ and the intermolecular and intramolecular H-bonds between the phosphonic acid groups at 2000–2300 cm^–1^.

To explore the differences in pore structure between the pristine polymer and the phosphonated polymer, we studied the nitrogen adsorption/desorption isotherms (Figure 2d). In the isotherms, a loss of desorption hysteresis in PIM-5F and emergence of almost ideal Langmuir isotherms in PIM-P were revealed, indicating a kinetic barrier to adsorption in PIM-5F, whereas the pore connection was better in PIM-P, which improved the efficiency of transporting substances. PIM-5F had a Brunauer–Emmett–Teller (BET) surface area of 538 m^2^ g^–1^, while PIM-P had a lower value of 395 m^2^ g^–1^. This might be due to the introduction of phosphonic acid groups that resulted in multiple interactions, including H-bonds and ion–dipole interactions between molecular chains, resulting in a highly rigid structure and denser packing of chains with a smaller average pore size. As shown in Figure 2e, the pore size distribution curves of the two offered additional evidence. The pore size peaks of >2 nm in PIM-P became weaker, while that of the smaller pores (<2 nm) increased, indicating a more concentrated distribution of pore sizes in the microporous region.

At room temperature, PIM-P in the dry state demonstrated good mechanical properties, with a stress of 47.2 MPa at maximum load and a strain of 18% at break (Table S2). These results indicated the ductility of the polymer, which might be attributed to the robust polymer backbone resulting in good mechanical properties and excellent film-forming properties of the resin (Figure S1). Compared with PIM-5F, dry PIM-P membrane was more brittle and was less resistant to bending. A similar phenomenon was also reported in previous studies,^41^ probably due to the introduction of phosphonic acids, which led to the formation of multiple interactions between and within molecular chains. The multiple interactions restricted the free movement of polymer chains (Figure 2f,g). The thermal stability of PIM-P was analyzed by thermogravimetric analysis (TGA) and differential scanning calorimetry (DSC). As shown in Figure S4, PIM-P exhibited good thermal stability, without weight loss, up to 350 °C. Based on the TGA curve, a continuous two-step weight loss of PIM-P was observed, which decomposed from 364 °C due to dephosphonation. The second stage of weight loss occurred from 460 °C, which might be related to the degradation or decomposition of the carbon–hydrogen skeleton. According to DSC results (Figure S5), no glass transition was detected up to 300 °C in PIM-P. Remarkably, the *T*_g_ was substantially higher than that of ionomers based on a PTFE backbone. The experimental results demonstrated that PIM-P had excellent structural stability at a high temperature.

**Figure 3:**
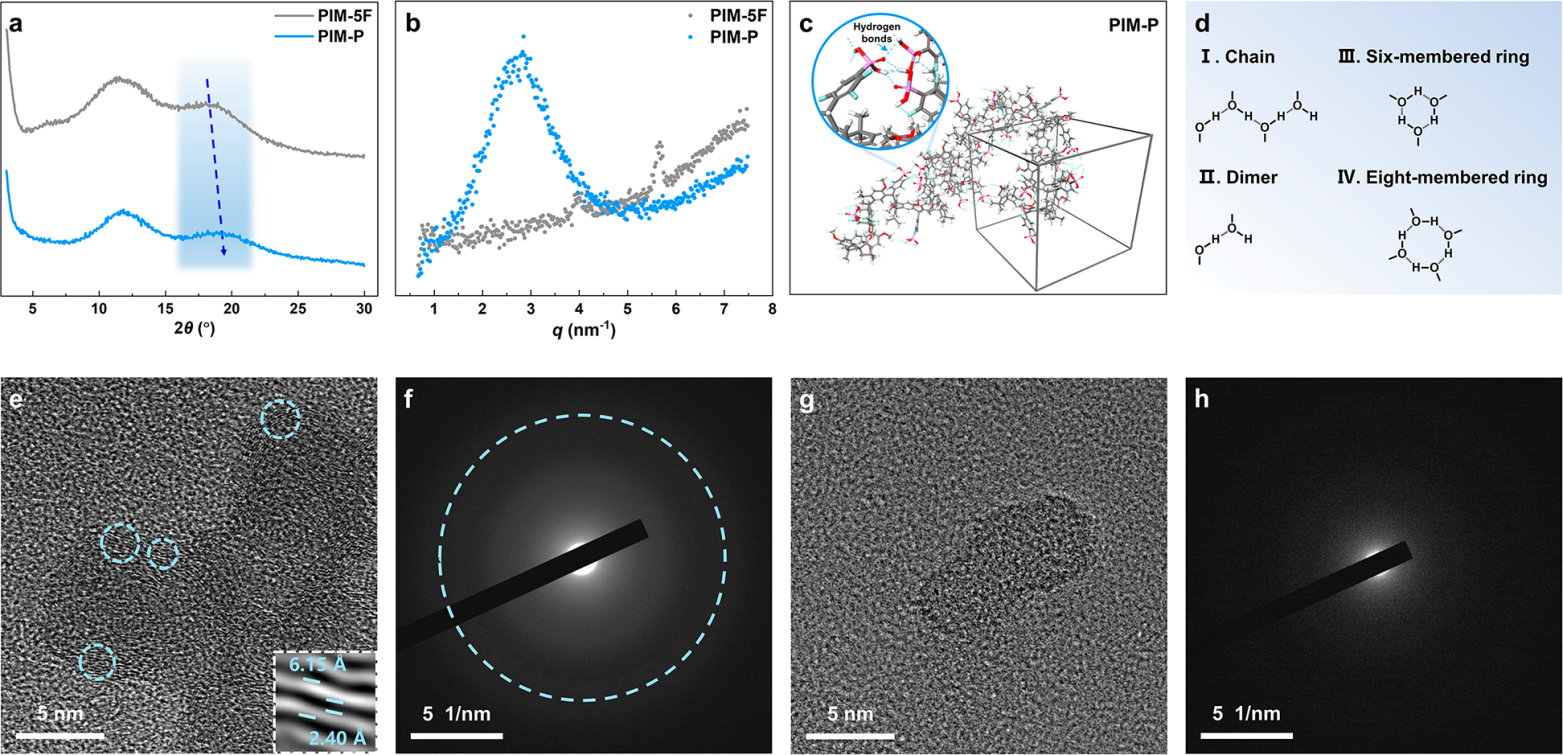
(a) XRD patterns of PIM-P and PIM-5F; (b) SAXS spectra of PIM-P and PIM-5F in dry state; (c) three-dimensional view of PIM-P in an amorphous periodic cell; (d) chemical structures of O–H···H aggregates in PIM-P; (e, f) HR-TEM image and SAED of PIM-P, respectively; (g, h) corresponding images of PIM-5F.

X-ray diffraction (XRD), small-angle X-ray scattering (SAXS), and high-resolution transmission electron microscopy (HR-TEM) were used to explore the nanoscale morphologies of phosphonated polymers. Both PIM-P and PIM-5F showed two obvious wide amorphous peaks in XRD profiles (Figure 3a), with some differences. The position of the 2θ = 18.4° peak of PIM-5F shifted to a higher 2θ value when phosphonic acid groups were introduced into the polymer backbone, and the domain spacing decreased from 4.82 to 4.57 Å according to Bragg’s law (2*d* sin θ = *nλ*). This was because that the presence of phosphonic acid groups enhanced intermolecular interactions and reduced the average chain spacing, which led to a tighter chain packing. Further, as shown in Figure 3b, the PIM-P ionomer in the dry state exhibited a characteristic SAXS profile: a distinct and intense peak at scattering vector *q* of 2.7 nm^–1^, which corresponded to the spatial correlation between ionic domains. The value of domain spacing was 2.33 nm (*d*_space_ = 2π/*q*), which also suggested short-distance order in PIM-P, due to the presence of phosphonic acid groups in PIM-P. Multiple interactions including H-bonds and dipole forces led to the assembly of side groups and regular stacking of polymer segments. Notably, the findings were consistent with the results of HR-TEM. As shown in Figure 3e, obvious light and dark changes were observed in the HR-TEM image of PIM-P, with a tendency toward a more orderly alignment over a short-range. The dark areas corresponded to the hydrophilic −PO_3_H_2_ while the bright domains reflected the hydrophobic regions, suggesting the formation of well-defined phase-separated structures in PIM-P. Further, PIM-P exhibited a regular orientation and orderly separation of the hydrophilic and hydrophobic regions. In Figure 3f, the selected-area electron diffraction (SAED) pattern showed broad diffraction rings, further corroborating the ordered arrangement in PIM-P, but with a shorter range. In contrast, no characteristic peaks appeared in the SAXS profiles of PIM-5F (Figure 3b) and no similar ordered arrangement was found in its HR-TEM images (Figures 3g and S7b). The ordered phase-separated structures in PIM-P contribute to the efficient transportation of gases and protons. A molecular model of PIM-P also showed the H-bonded chain interactions, which led to the aggregation of phosphonic acid groups (Figure 3c). Phosphonic acid groups act as both donors and acceptors of H-bonds and form several types of hydrogen-bonded O–H···O aggregates including chains, dimers, and rings (Figure 3d).^42,43^ These aggregates constituted a continuous network of H-bonds between molecular chains. Due to the presence of intermolecular H-bonds, the internal rotation of molecular segments was restricted, resulting in a partially ordered structure. Although they limited the free movement of chains, they greatly improved mass transport channels and the stability of the polymer pore structure.

Water uptake and swelling ratio were evaluated from 30 to 80 °C. As expected, PIM-P showed only a minor increase in both water uptake and swelling ratio with the increase of temperature (Figure S8), due to the higher rigidity of the backbone and the morphological structure with well-connected hydrophilic nanochannels. Meanwhile, owing to the high-molecular-weight, rigid, and contorted polymer backbone, which limited the ion diffusion through chains, the proton conductivity of PIM-P was 74 mS cm^–1^ at 160 °C under humidified conditions (Figure S9, S10). Fortunately, a high proton conductivity was not essential for the ionomeric binder.^29^ Oxidative stability is one of the important parameters determining the performance of ionomers used in HT-PEMFCs. It was tested by immersing PIM-P into Fenton’s reagent at 80 °C. No obvious change in structure was detected by ^1^H NMR spectroscopy during a period of 144 h (Figure S11). These results suggested that PIM-P with an ether-free backbone had good chemical stability, which resisted the attack of hydroxyl radicals.

**Figure 4:**
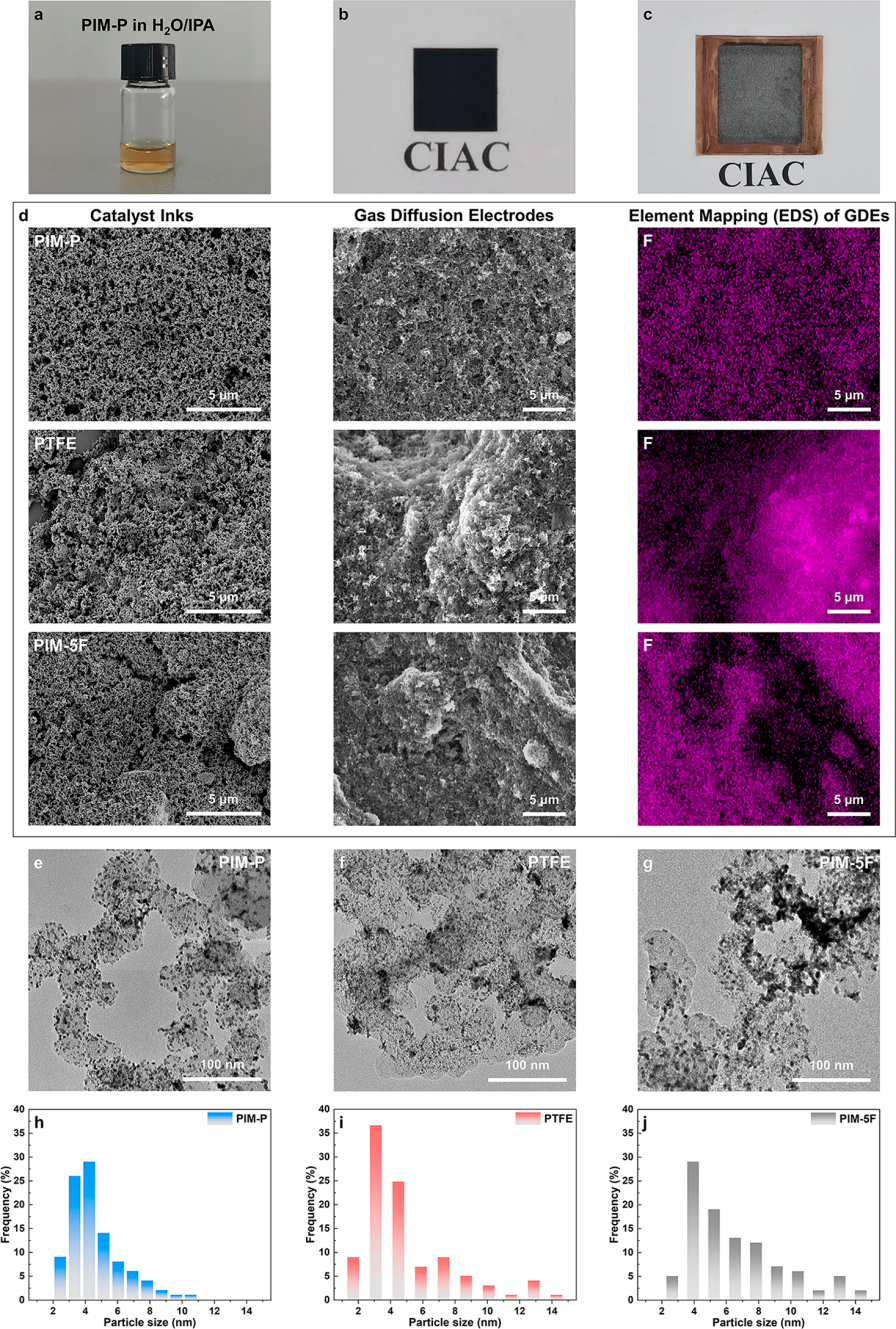
(a) Photographs of PIM-P solution, (b) GDE and (c) MEA with PIM-P ionomer; (d) SEM images of catalyst inks, SEM images, and corresponding EDS elemental mapping of GDEs; (e–g) TEM images of Pt/C coated with different catalyst binders and (h–j) corresponding particle size distributions.

The solubility in different solvents is summarized in Table S4. In addition to dissolving in DMAc, DMSO, and other high boiling point solvents, PIM-P also showed good solubility in polar protic solvents, such as alcohols. In particular, it exhibited excellent solubility at a concentration of 5 wt %/v in 25 vol %/75 vol % and 50 vol %/50 vol % water (H_2_O)/isopropanol (IPA) mixtures (Figure S12), which facilitated the even dispersion of PIM-P in the catalyst ink slurry with the H_2_O/IPA dispersant. Scanning electron microscopy (SEM) was conducted to analyze the properties of catalyst inks and gas diffusion electrodes (GDEs) using different catalyst binders with the same amount (20 wt %) and microstructures presented in Figure 4d. Larger lumps corresponding to bulky catalyst and binder agglomerates were observed in both inks and GDEs with PTFE and PIM-5F. However, no large aggregates were found, and a more uniform dispersion was observed in the SEM images with PIM-P binder. Further, it showed the existence of secondary pores in SEM images with PIM-P ionomer. The dispersion of binders was also confirmed via energy-dispersive spectroscopy (EDS) elemental analysis. TEM images of Pt/C coated with different catalyst binders are shown in Figure 4e–g, and the Pt particle size distributions are shown in the illustrations. Obviously, the Pt particle coated with PIM-P ionomer shows the narrowest size distribution, and larger agglomerates of Pt particles are observed in the TEM images with PTFE and PIM-5F binders.^44,45^ These results indicate that the PIM-P ionomer facilitates the uniform distribution of the catalyst particles. It could be predicted that PIM-P promoted better coating on the surface of the Pt/C particles and the formation of an appropriate TPB, which was essential to efficient mass transport in cells.^46^

**Figure 5:**
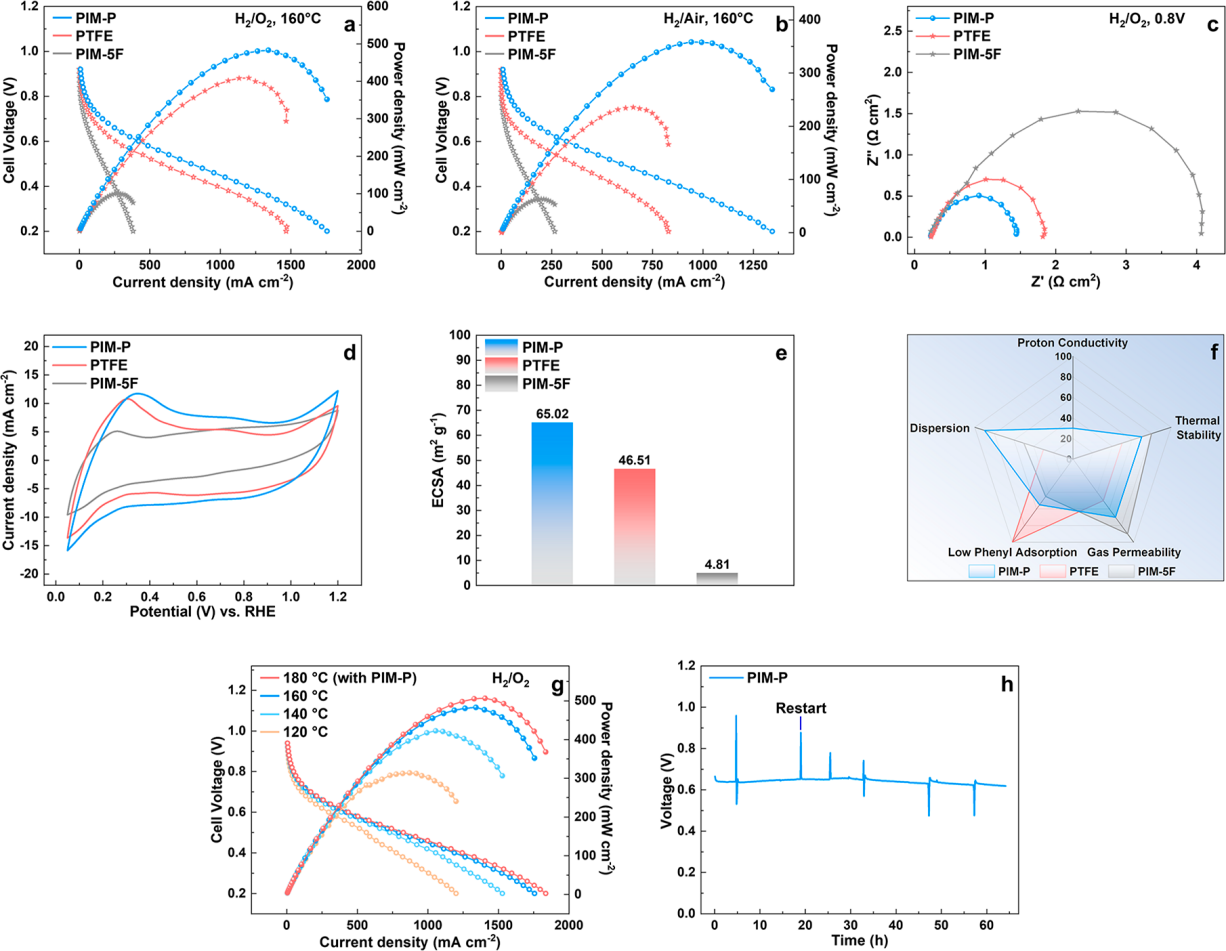
Polarization and power density curves of MEAs with different kinds of catalyst binders at 160 °C under (a) H_2_/O_2_ condition and (b) H_2_/air condition, respectively; (c) Nyquist plots of MEAs which were obtained at 0.8 V under H_2_/O_2_ condition; (d) cyclic voltammograms (CV) for Pt/C catalyst with different binders; (e) corresponding ECSA values; (f) property spider charts of different materials to be used as electrode binder; (g) polarization and power density curves of the MEA with PIM-P ionomer at a series of temperatures under H_2_/O_2_ condition; (h) in situ durability of H_2_/O_2_ HT-PEMFC based on PIM-P testing under a constant current density of 150 mA cm^–2^ at 160 °C.

In addition to electrode binder materials, the PEMFC performance of MEAs constituting the same components was also compared under H_2_/O_2_ and H_2_/air conditions at 160 °C, respectively. The polarization and power density curves are shown in Figure 5a,b. They showed a similar trend when comparing the results under both H_2_/O_2_ and H_2_/air conditions, with the MEA with the PIM-P ionomer showing the best fuel cell performance in both cases. At low current density, the drop in polarization curves was attributed to a loss of activation. PIM-P MEA showed a minimum voltage drop due to stable proton conductivity. The MEAs with PIM-P and PTFE binders demonstrated a similar linear decrease in slopes during the subsequent drop of polarization curves, which was attributed to similar ohmic loss. At high current density, the curves of MEA with PTFE and PIM-5F binders showed obvious mass transport loss. The high mass transport resistance of the MEA with PTFE binder might be attributed to the insolubility of binder in catalyst inks, which decreased the uniform distribution and showed no proton conduction. The voltage drop of MEAs with PIM-5F binders might be attributed to the films formed on the catalyst sites because of the cover effect, resulting in the low gas permeability of binders and limited power density of MEAs. Especially, compared with PTFE, the peak power density of the MEA with PIM-P ionomer was 18% greater than that of PTFE under H_2_/O_2_ condition and 52% under H_2_/air conditions. The higher performance could be attributed to the intrinsic microporous structure and proton-conductive functionality of PIM-P. To further verify the difference in cell resistance with different binders, electrochemical impedance spectroscopy (EIS) measurements were conducted at 0.8 V (Figure 5c) and 1000 mA cm^–2^ (Figure S13), respectively. The diameter of the plot representing the MEA with the PIM-P binder was the smallest, suggesting that the GDEs had the lowest mass transport resistance. It can be found that the MEA with the PIM-P ionomer has the lowest resistance at both high and low current densities. It can be found that the MEA with the PIM-P ionomer has the lowest resistance at both high and low current densities. These results also fit with their polarization and power density curves, with the lower resistance leading to a higher single cell performance. As shown in Figure 5d,e, the electrochemical active surface area (ECSA) of different catalyst binder systems was calculated from their cyclic voltammograms. The catalyst with the PIM-P ionomer had the highest ECSA value of 65.02 m^2^ g^–1^ Pt. This demonstrated that the unique proton conductivity and microporous structure of PIM-P could enhance the utilization of the Pt/C catalyst.

Further, the power density of the MEA with PIM-P binder showed a continuous increase with the increase of temperature due to higher proton conductivity through the O-PBI membrane and reached 506.6 mW cm^–2^ at 180 °C without external humidification and backpressure (Figure 5g). As shown in Figure 5h, an in situ durability test revealed that the present HT-PEMFC based on the PIM-P binder and an O-PBI/phosphoric acid (PA) membrane exhibited good stability under a current density of 0.15 A cm^–2^ at 160 °C, and no voltage loss occurred within the 65 h of the durability test. Further, the MEA performance was enhanced during 30 h, which could be explained by the activation of CLs. Nonetheless, a slight voltage loss occurred in the cell after 30 h, with a decay rate of 1.07 mV h^–1^. These results further demonstrated that the activation of the MEA with the PIM-P binder at a constant current density contributed to the improved performance of the CLs.

As shown in Figure 5f, the higher performance of the MEA with the PIM-P ionomer compared with other MEAs could be attributed to the stable proton conductivity and high gas permeability (Table S6), which facilitated the proton conductivity of the binder while creating a robust gas transmission channel. In addition, the ordered structure based on the −PO_3_H_2_ assembly between molecular chains also significantly improved the efficiency and stability of mass transport of CLs. Besides, PIM-P exhibited excellent solubility in H_2_O/IPA mixtures, which contributed to the dispersion of ionomer in the catalyst ink and the formation of ultrathin coatings around Pt/C particles. These results suggested that a rational design of the binder structure reduces the cell resistance to improve the overall performance of fuel cells. In brief, compared with state-of-the-art commercial binders, PIM-P is a potential next-generation binder for PEMFC applications.

## Conclusion

In summary, a novel phosphonated ionomer with both intrinsic microporous structure and proton-conductive functionality was designed as the catalyst binder of HT-PEMFCs to improve the mass transport of electrodes. The pore structure of PIM-P was facilitated by multiple interactions, including intermolecular H-bonds and dipole forces. Notably, because of group assembly based on the aggregates of phosphonic acid groups, PIM-P showed a tendency for orderly alignment over a short range, resulting in the formation of a fast transport channel in the ionomer that contributed to the development of ideal mass transport pathways. Meanwhile, the synthesized ionomer displayed good dispersibility in catalyst inks, great structure stability, and high pore stability as well as the lower phenyl content of the backbone, which naturally alleviated phenyl adsorption. In contrast, the MEA with PIM-P showed lower mass transport resistance with the peak power density reaching 506.6 mW cm^–2^, which was 18–379% greater than that of other commercial or porous binders at 160 °C under H_2_/O_2_ conditions. The findings suggest that a comprehensive analysis of gas permeability, proton conductivity, and interfacial compatibility is essential to improve the performance of ionomers and provided a new idea for improving mass transport at electrodes from the perspective of structural design rather than complex processes. Further, the study emphasizes the crucial role of order structure for anhydrous proton-conducting in PIMs and opens a new method to construct high-performance ionomers by adjusting and modifying the microporous structure of PIMs.

## Experimental Section

### Materials

Tris(trimethylsilyl) phosphite (TSP, 95.0%, TCI), Pt/C catalyst (40 wt % Pt, HTP040, HESEN), carbon paper (TGP-H-060, TORAY), and poly(tetra fluoroethylene) (PTFE) emulsion (60 wt %, D-210C, DAIKIN) were purchased from the respective companies. All solvents were purchased from Sinopharm Group Chemical Reagent Co. and used as received.

### Synthesis of PIM-P

Experimental details for the preparation of PIM-5F can be found in ref (47). Under a N_2_ atmosphere, PIM-5F (2.0581 g, 4 mmol), TSP (5.9708 g, 20 mmol), and dimethylacetamide (DMAc) (15 mL) were added to a 50 mL three-neck round-bottom flask. The reaction mixture was then heated to 190 °C and allowed to react for 12 h. Once cooled to room temperature, the solution was poured into deionized water and stirred overnight. The precipitated polymer was vacuum filtered and hydrolyzed with refluxing 1 M HCl twice to ensure the complete hydrolysis to phosphonic acid. After this time the polymer was filtered by vacuum filtration and rinsed with deionized water. PIM-P was dried in vacuo overnight, yield 94%.

### MEA Preparation

Catalyst inks were composed of 40 wt % Pt/C, ionomer solution, water (H_2_O), and isopropanol (IPA). PIM-P was dissolved into 25 v%/75 v% H_2_O/IPA mixtures at a 5 wt %/vol % concentration to prepare the ionomer solution. Before spraying, the catalyst inks were employed with ultrasonication for 30 min to ensure good dispersion and then were painted onto the carbon paper to obtain gas diffusion electrodes (GDEs). The catalyst loading on each electrode was 1 mg cm^–2^ Pt loading, and the content of binder in the catalyst layers (CLs) was controlled at 20 wt %. MEAs were prepared by sandwiching the commercial O-PBI/phosphoric acid (PA) membrane (40 μm) between two pieces of GDEs.

## Supporting Information Available

Detailed experimental descriptions, ^1^H NMR, FT-IR, mechanical properties, TGA, DSC, solubility, IEC, water uptake, swelling ratio, conductivity, oxidative stability, and gas permeability (PDF)
