# Supplementary material for: Phosphonated Ionomers of Intrinsic Microporosity with Partially Ordered Structure for High-Temperature Proton Exchange Membrane Fuel Cells
Source: ACS Cent Sci. 2023 Mar 16;9(4):733–41. doi: 10.1021/acscentsci.3c00146 (PMC10141605; doi:10.1021/acscentsci.3c00146)
Supplement: Supplementary file 1 — oc3c00146_si_001.pdf [file oc3c00146_si_001.pdf]

Supporting Information

Phosphonated Ionomers of Intrinsic Microporosity with Partially Ordered Structure for High-Temperature Proton Exchange Membrane Fuel Cells

Xi Sun, [a,b] *Jiayu Guan,* [a,b] *Xue Wang,* [a,b] *Xiaofeng Li,* [a,b] *Jifu Zheng,* [a*] *Shenghai Li,* [a,b] *and Suobo Zhang* [a,b*]

[a]Key Laboratory of Polymer Ecomaterials, Changchun Institute of Applied Chemistry, Chinese Academy of Sciences, Changchun 130022, China.

[b]University of Science and Technology of China, Hefei 230026, China.

[*]Correspondence should be addressed to J. Z. (jfzheng@ciac.ac.cn) and S. Z. (sbzhang@ciac.ac.cn)

This file includes:

24 pages,

13 figures (Figure S1 to S13),

and 6 tables (Table S1 to S6).

Contents

Experimental Section S3

Scheme S1. Synthetic pathway to PIM-P S3

Characterization and Measurements S3

Supplementary results S7

Table S1. Molecular weight of PIM-5F S7

Figure S1. A photograph of PIM-P membrane S8

Figure S2. ^{1}H NMR spectrum of PIM-P (DMSO-d_6) S9

Figure S3. FT-IR spectra of PIM-5F and PIM-P membranes S10

Table S2. Mechanical properties of PIM-P membrane in dry state S11

Figure S4. TGA curve of PIM-P under N_2 atmosphere S12

Figure S5. DSC curve of PIM-P S13

Figure S6. Three-dimensional view of PIM-5F in an amorphous periodic cell S14

Figure S7. TEM images of PIM-P and PIM-5F S15

Table S3. IEC of PIM-P in acid form S16

Figure S8. Temperature-dependent water uptake and swelling ratio of PIM-P S17

Figure S9. Proton conductivity of PIM-P membrane from 30°C to 80°C in the fully hydrated state S18

Figure S10. Proton conductivity of PIM-P membrane from 90°C to 160°C under humidified condition S19

Figure S11. ^{1}H NMR spectra of PIM-P before and after soaking in Fenton's reagent at 80°C S20

Table S4. The solubility of PIM-P and PIM-5F S21

Figure S12. Solubility of PIM-P at a concentration of 5 % wt/v in H_2O/IPA mixtures S21

Table S5. Phosphoric acid (PA) doping level of the O-PBI membrane S22

Table S6. The permeability (*P*) of different gases for PIM-P and PIM-5F S23

Figure S13. Nyquist plots of MEAs at 1000 mA cm^{-2} S24

Experimental Section

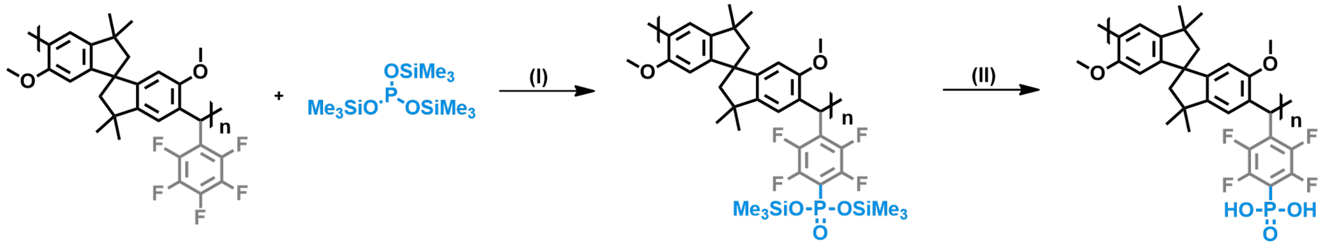

Scheme S1. Synthetic pathway to the phosphonated polymer of intrinsic microporosity (PIM-P): (I) phosphonation (DMAc, 190°C, 12 h); and (II) hydrolysis (1 M HCl, 110°C, 12 h).

Characterization and Measurements

^{1}H nuclear magnetic resonance (NMR), ^{19}F NMR, and ^{31}P NMR spectra were obtained on a Bruker AV 500 spectrometer at 500 MHz using DMSO-d$_6$ and $CDCl_3$ as solvent. Fourier transform infrared (FT-IR) spectra were obtained using a Bruker VERTEX 70v FT-IR spectrometer with a ZnSe/Diamond ATR accessory. Gel permeation chromatography (GPC) with POLYMER LABORATORIES-GPC120 was used to determine the molecular weight of PIM-5F at 30°C, while tetrahydrofuran (THF) was used as eluent. The Brunauer–Emmett–Teller (BET) surface area was determined by measuring the nitrogen gas adsorption/desorption isotherms of the polymers at 77 K and the pore size distribution was calculated with density functional theory (DFT). Tensile stress-strain behavior of membranes was measured with an Instron-1121 mechanical testing instrument at a crosshead speed of 2 mm min^{-1}. Modelling structure of homopolymers were constructed using the Amorphous Cell module in Material Studio (Accelrys Inc, CA, USA). Thermogravimetric analysis (TGA) was measured with a PerkinElmer TGA-2 thermogravimetric analyzer with a rate of 10°C min^{-1} under continuous nitrogen flow. Differential scanning calorimetry (DSC) analysis was tested using a PerkinElmer DSC-7 at a temperature range of -50°C to 300°C and a heating rate of 10°C min^{-1} under N_2 atmosphere. The X-ray diffraction (XRD) measurements were taken on a Rigaku MiniFlex 600 (Cu K$_\alpha$ radiation, λ = 1.5405 Å) at a scanning rate of 10° min^{-1} (2θ from 2° to 30°). Small-angle X-ray scattering (SAXS) was carried out on a

SAXSpoint 2.0 apparatus (Anton Paar). The membranes were irradiated by X-ray (CuK$_\alpha$, λ_i = 1.542 Å) with 40 kV and 50 mA and the range of scattering vectors explored was 0.6 to 7.5 nm^{-1}. High-resolution transmission electron microscope (HR-TEM) analysis was performed on a JEOL JEM-F200 instrument operated at an accelerating voltage of 200 kV. The samples used for TEM characterization were in membrane form on copper grids. Scanning electron microscopy (SEM) spectra were measured on a Zeiss Merlin FE-SEM.

The acid-base titration method was used to determine the experimental ion exchange capacity (IEC). The PIM-P sample membranes in the H^+ form were first immersed in 30 mL of 1 M NaCl (aq) and equilibrated for 24 h to release the H^+ ions. These solutions were then back-titrated with 0.01 M NaOH solution using phenolphthalein as an indicator. The final IEC (mequiv g^{-1}) results were obtained from the following equation:

$$\mathrm{IEC} = \frac{(30 - V) \times 0.01}{M}$$

where V is the volume (mL) of NaOH solution that was used at the end point of the titration and M is the weight (g) of the measured membrane.

The membrane was cut into cuboid of 4.5 cm × 1.0 cm × 40 μm for determination of water uptake (WU) and swelling ratio (SR). To measure wet mass (M_w) and wet length (L_w), the membrane was immersed into deionized water at RT overnight and then moved into different temperatures of deionized water for 1 h. After that, the membrane was removed from the water and the residual water was quickly wiped from the surface using a filter paper. To measure dry mass (M_d) and dry length (L_d), the same sample was dried in a vacuum oven at 80°C for 12 h. The mass and length were obtained using an analytical balance and a vernier caliper, respectively. The formulas for WU and SR are as follows:

$$\mathit{Water\ uptake}\ (\%) = \frac{M_w - M_d}{M_d} \times 100\ \%$$

$$\mathit{Swelling\ ratio}\ (\%) = \frac{L_w - L_d}{L_d} \times 100\ \%$$

In-plate proton conductivity (σ, S cm^{-1}) of PIM-P membrane (4.0 cm × 1.0 cm × 40 μm) was measured using a four-point probe method with alternating current impedance on an electrochemical workstation (BioLogic VSP) and the frequency ranged from 10 Hz to 100 kHz. The conductivity measurements were carried out with the test system immersed in liquid water or under humidified condition. The range of test temperature was 30°C to 80°C and 90°C to 160°C, respectively. And at each temperature, the system was equilibrated for at least 1 h before measuring the resistance value. Conductivity was calculated by using following formula:

$$\sigma = \frac{D}{RA}$$

where σ is proton conductivity, D is the distance between two adjacent probes, R is the measured resistance value and A is the cross-sectional area of the membrane.

In this work, the oxidative stability of PIM-P was tested by immersing the membrane into Fenton's reagent (3% H_2O_2 aqueous solution containing 2 ppm Fe^{2+}) at 80°C. Every once in a while, a small part of the membrane sample was taken out to run ^1H NMR spectrum to investigate the changes of structure.

To evaluate the performance of PIM-P binder, the membrane electrode assemblies (MEAs) were then assembled in a single cell fixture with an active area of 9.0 cm^2 (3.0 cm × 3.0 cm) for high-temperature proton exchange membrane fuel cells (HT-PEMFCs) test. Testing conditions: anode/cathode: 40% Pt/C (Pt loading: 1.0 mg cm^{-2}), binder (20 wt%); HT-PEM: PA-doped O-PBI. And electrochemical characterization was initiated by running polarization and power density curves, electrochemical impedance spectroscopy, cyclic voltammograms and in-situ durability. Polarization and power density curves were measured using a fuel cell test station (Dongfang Electric Corporation Limited) with no humidification or backpressure. The single cell was operated under ambient pressure from 120°C to 180°C with dry H_2 at 250 sccm and O_2 (or air) at 500 sccm fed into anode and cathode, respectively. Prior to the test, the cell was active at a constant voltage of 0.6 V for at least 1 h until the current stable. And then, the polarization curves were recorded. Electrochemical impedance spectroscopy (EIS) of HT-PEMFCs MEAs was measured

by a Biologic electrochemical workstation (Biologic VSP, France). In situ EIS analysis was performed by sweeping frequencies from 100 Hz to 1 MHz at 160°C after cell stabilization about 30 mins. Cyclic voltammetry (CV) was conducted from 0.05 V to 1.2 V at a sweep rate of 50 mV s^{-1} and room temperature with H_2 at 250 sccm and N_2 at 500 sccm fed into anode and cathode, respectively. In-situ durability of H_2/O_2 HT-PEMFC based on PIM-P binder testing under a constant current density of 150 mA cm^{-2} at 160°C with a 250/500 sccm anode/cathode flow rate.

The gas permeabilities were determined at 35°C and 100 psi upstream pressure using the constant-volume/variable-pressure method. The permeability (*P*) was calculated by using following formula:

$$P = 10^{10} \times \frac{V \times l}{A \times R \times T \times p} \times \frac{dp}{dt}$$

The unit for permeability values (*P*) is Barrer (1 Barrer = 10^{-10} cm^3•(STP)•cm/(s•cm^2•cmHg)). Where *V* (cm^3) is the calibrated downstream volume, *l* (cm) is membrane thickness, *A* (cm^2) is the authentic membrane area, *R* is general gas constant (0.278 cm^3•cmHg/cm^3(STP)•K), *T* (K) is the operating temperature (K), *p* is the upstream pressure, *dp/dt* is the permeate side stable flow rate.

Supplementary results

Table S1. Molecular weight of PIM-5F. (Eluent: tetrahydrofuran (THF); test temperature: 30 °C.)

**Sample**	M_n (kDa)	M_w (kDa)	PDI (Polymer dispersity index)
**PIM-5F**	66	119	1.8

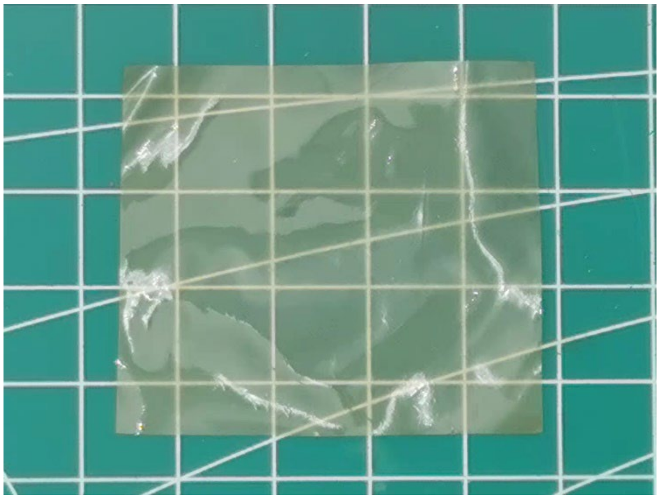

Figure S1. A photograph of PIM-P membrane.

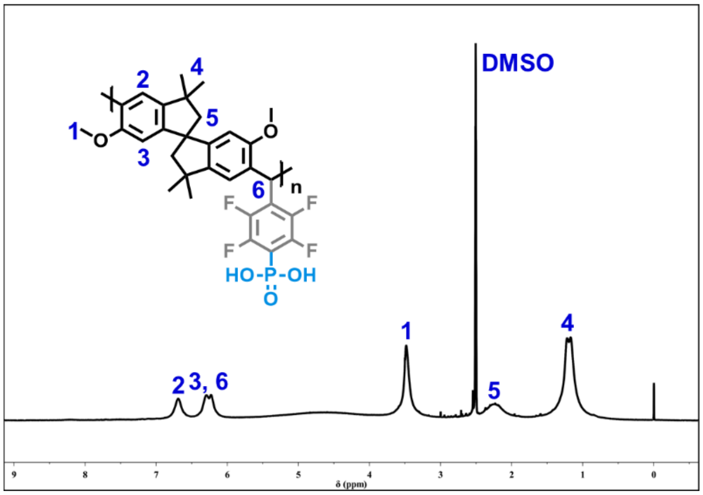

Figure S2. ^{1}H NMR spectrum of PIM-P (DMSO-d_6).

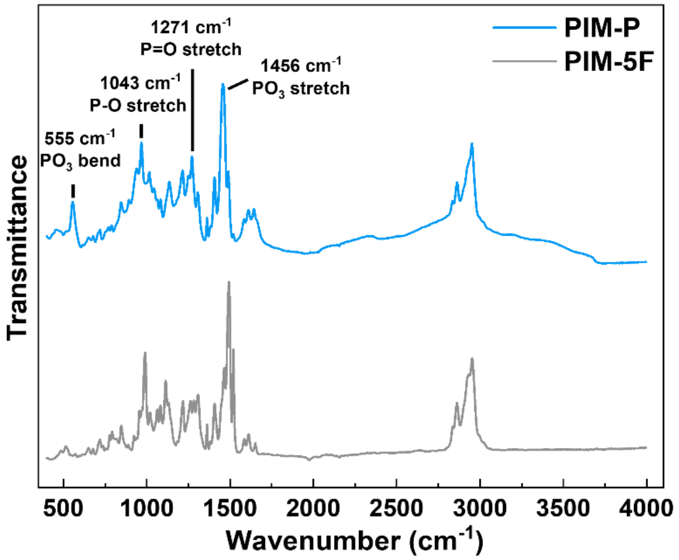

Figure S3. FT-IR spectra of PIM-5F and PIM-P membranes.

Table S2. Mechanical properties of PIM-P membrane in dry state.

**Sample**	tensile strength (MPa)	elongation at break (%)	Young’s modulus (MPa)
**PIM-P**	47.2	18	987

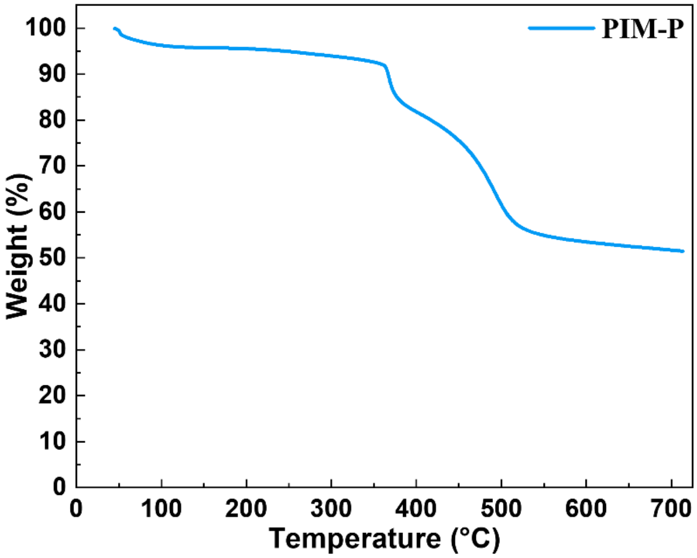

Figure S4. TGA curve of PIM-P under N_2 atmosphere.

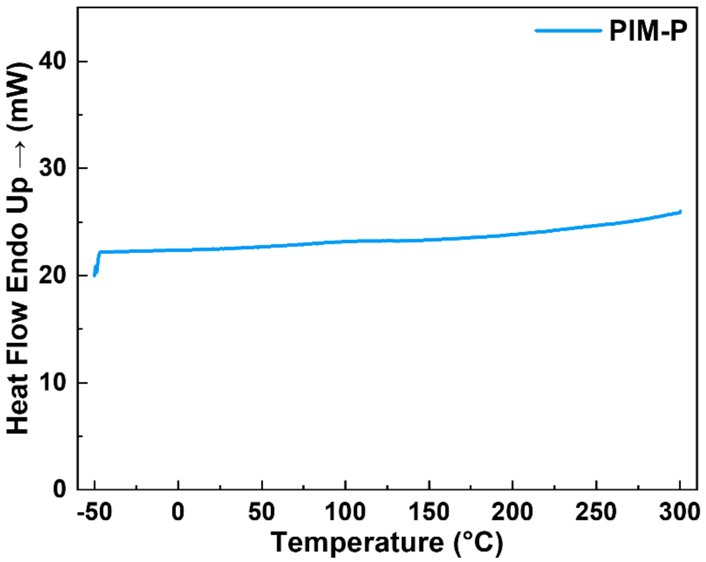

Figure S5. DSC curve of PIM-P.

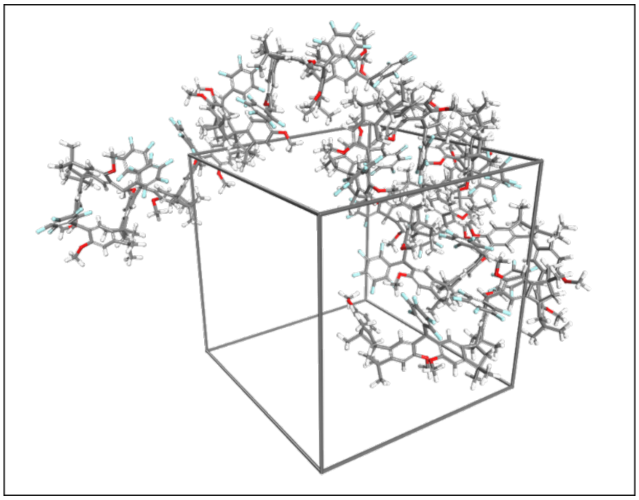

Figure S6. Three-dimensional view of PIM-5F in an amorphous periodic cell.

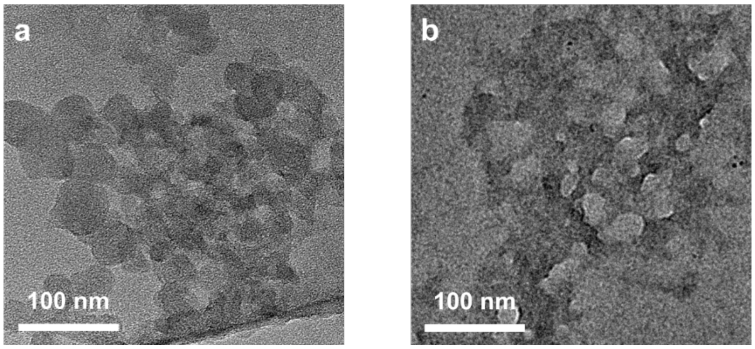

Figure S7. TEM images of (a) PIM-P and (b) PIM-5F.

Table S3. IEC of PIM-P in acid form.

**IEC (mequiv g^{-1})**	titrated
**PIM-P**	1.68

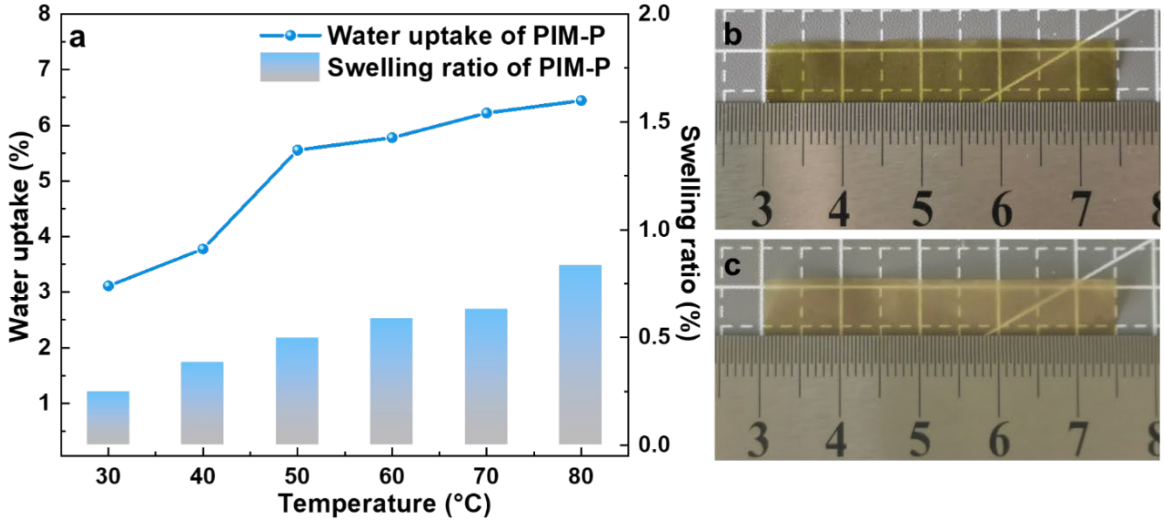

Figure S8. (a) Temperature-dependent water uptake and swelling ratio of PIM-P; (b) A photograph of PIM-P membrane in dry state; (c) A photograph of PIM-P membrane after soaking in water at 80°C for 24 h.

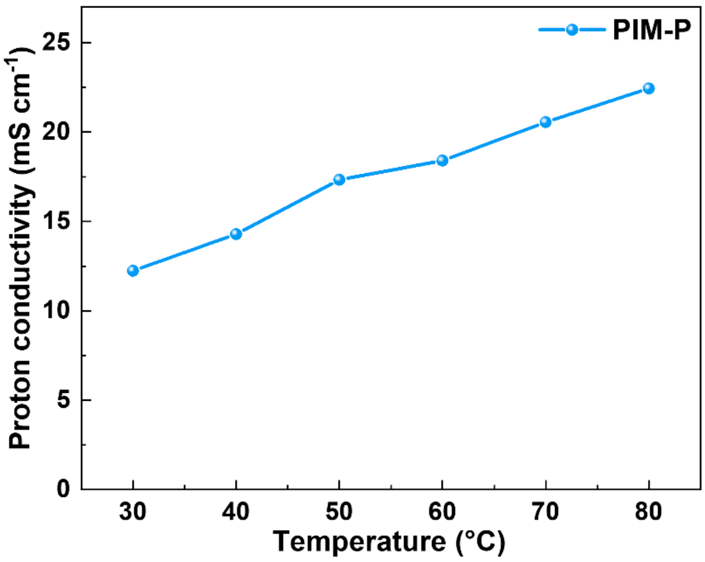

Figure S9. Proton conductivity of PIM-P membrane from 30°C to 80°C in the fully hydrated state.

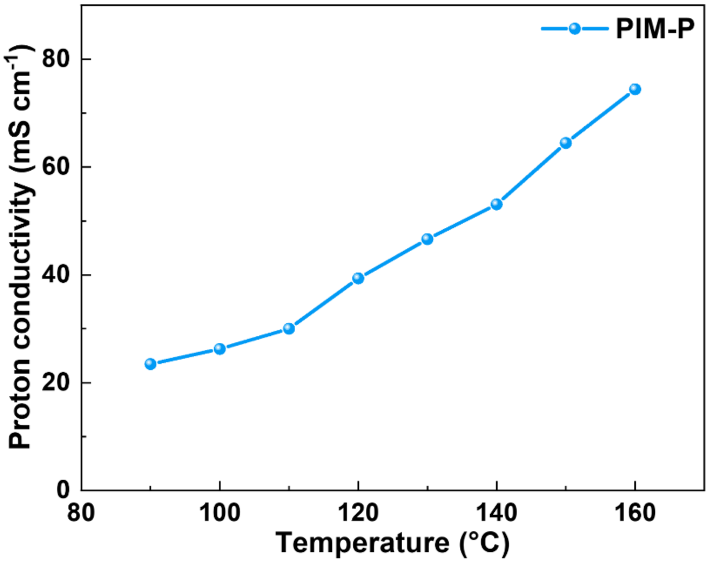

Figure S10. Proton conductivity of PIM-P membrane from 90°C to 160°C under humidified condition.

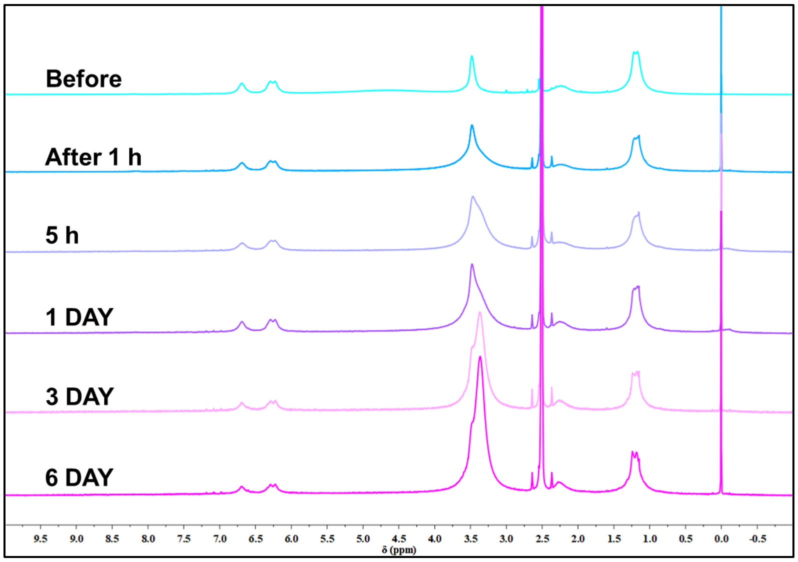

Figure S11. ^{1}H NMR spectra of PIM-P before and after soaking in Fenton's reagent at 80°C.

Table S4. The solubility of PIM-P and PIM-5F. Solubility of polymers was tested by adding 25 mg of polymer in 0.5 mL of solvent (5 wt/v %). (++) polymer is completely soluble. (+) polymer is partially soluble. (-) polymer is insoluble.

Sample	DMF	DMAc	DMSO	NMP	MeOH	EtOH	IPA	25 v%/75 v% H_2O/IPA	50 v%/50 v% H_2O/IPA	75 v%/25 v% H_2O/IPA	CH_2Cl_2
**PIM-P**	+	++	++	++	+	++	+	++	++	-	-
**PIM-5F**	+	++	+	++	-	-	-	-	-	-	++

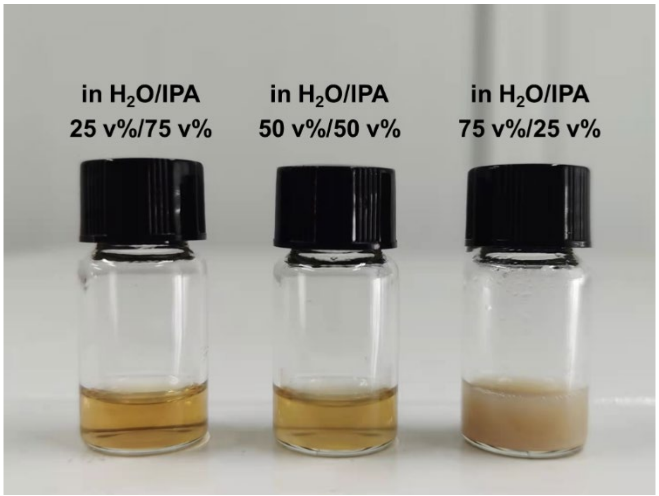

Figure S12. Solubility of PIM-P at a concentration of 5% wt/v in H_2O/IPA mixtures.

Table S5. Phosphoric acid (PA) doping level of the O-PBI membrane.

**Membrane**	O-PBI
**Phosphoric acid (PA) doping content (%)**	229.37%
**Acid doping level (ADL)**	9.30
**Area swelling (%)**	59.66%
**Volume swelling (%)**	139.82%
**Phosphoric acid (PA) doping density (ADL/Volume swelling)**	0.07
**Proton conductivity at 160°C (mS cm^{-1})**	42.14 mS cm^{-1}

Table S6. The permeability (*P*) of different gases for PIM-P and PIM-5F under dry condition.

***P* (Barrer)**	H_2	O_2
**PIM-P**	35.00	4.21
**PIM-5F**	1635.9	464.9

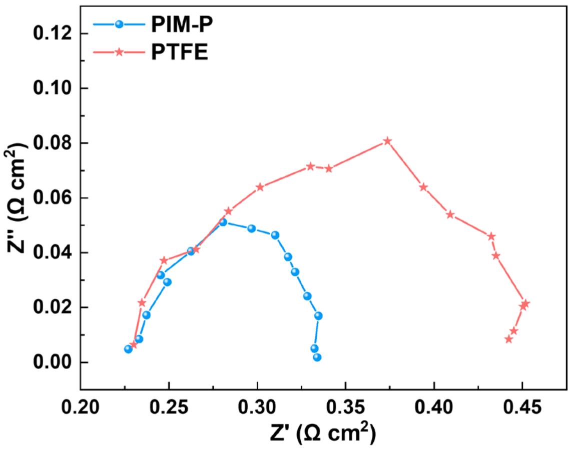

Figure S13. Nyquist plots of MEAs which were obtained at 160°C with a frequency range of 100 Hz to 1 MHz at 1000 mA cm^{-2} under H_2/O_2 condition.
